# Microbial Culture in Minimal Medium With Oil Favors Enrichment of Biosurfactant Producing Genes

**DOI:** 10.3389/fbioe.2020.00962

**Published:** 2020-08-11

**Authors:** W. J. Araújo, J. S. Oliveira, S. C. S. Araújo, C. F. Minnicelli, R. C. B. Silva-Portela, M. M. B. da Fonseca, J. F. Freitas, K. K. Silva-Barbalho, A. P. Napp, J. E. S. Pereira, M. C. R. Peralba, L. M. P. Passaglia, M. H. Vainstein, L. F. Agnez-Lima

**Affiliations:** ^1^Laboratório de Biologia Molecular e Genômica, Departamento de Biologia Celular e Genética, Centro de Biociências, Universidade Federal do Rio Grande do Norte, Natal, Brazi; ^2^INESC-ID/IST – Instituto de Engenharia de Sistemas e Computadores/Instituto Superior Técnico, Universidade de Lisboa, Lisbon, Portugal; ^3^Laboratório de Fungos de Importância Médica e Biotecnológica, Centro de Biotecnologia, Universidade Federal do Rio Grande do Sul, Porto Alegre, Brazil; ^4^Laboratório de Química Analítica e Ambiental, Departamento de Química, Instituto de Química, Universidade Federal do Rio Grande do Sul, Porto Alegre, Brazil; ^5^Laboratório de Genética Molecular Vegetal, Departamento de Genética, Instituto de Biociência, Universidade Federal do Rio Grande do Sul, Porto Alegre, Brazil

**Keywords:** biodegradation, biotechnology, petrochemical waste, consortia, metagenomics, production water, biosurfactant

## Abstract

The waste produced by petrochemical industries has a significant environmental impact. Biotechnological approaches offer promising alternatives for waste treatment in a sustainable and environment-friendly manner. Microbial consortia potentially clean up the wastes through degradation of hydrocarbons using biosurfactants as adjuvants. In this work, microbial consortia were obtained from a production water (PW) sample from a Brazilian oil reservoir using enrichment and selection approaches in the presence of oil as carbon source. A consortium was obtained using Bushnell-Haas (BH) mineral medium with petroleum. In parallel, another consortium was obtained in yeast extract peptone dextrose (YPD)-rich medium and was subsequently compared to the BH mineral medium with petroleum. Metagenomic sequencing of these microbial communities showed that the BH consortium was less diverse and predominantly composed of *Brevibacillus* genus members, while the YPD consortium was taxonomically more diverse. Functional annotation revealed that the BH consortium was enriched with genes involved in biosurfactant synthesis, while the YPD consortium presented higher abundance of hydrocarbon degradation genes. The comparison of these two consortia against consortia available in public databases confirmed the enrichment of biosurfactant genes in the BH consortium. Functional assays showed that the BH consortium exhibits high cellular hydrophobicity and formation of stable emulsions, suggesting that oil uptake by microorganisms might be favored by biosurfactants. In contrast, the YPD consortium was more efficient than the BH consortium in reducing interfacial tension. Despite the genetic differences between the consortia, analysis by a gas chromatography-flame ionization detector showed few significant differences regarding the hydrocarbon degradation rates. Specifically, the YPD consortium presented higher degradation rates of C12 to C14 alkanes, while the BH consortium showed a significant increase in the degradation of some polycyclic aromatic hydrocarbons (PAHs). These data suggest that the enrichment of biosurfactant genes in the BH consortium could promote efficient hydrocarbon degradation, despite its lower taxonomical diversity compared to the consortium enriched in YPD medium. Together, these results showed that cultivation in a minimal medium supplemented with oil was an efficient strategy in selecting biosurfactant-producing microorganisms and highlighted the biotechnological potential of these bacterial consortia in waste treatment and bioremediation of impacted areas.

## Introduction

During oil production, processing, and storage operations, large volumes of waste are generated such as oily sludge, waste rock, and production water (PW), which exhibit highly toxic hydrocarbon concentrations and pose a serious environmental risk, demanding treatment before disposal (Neff et al., 2011; Cordes et al., 2016; Al-Ghouti et al., 2019). PW reinjection is an oil recovery method that increases oil production by 15–25% (Shibulal et al., 2014). Despite this reuse, contaminated water continues to be produced, requiring treatment before disposal (Al-Ghouti et al., 2019).

Therefore, the growth in oil production increases the demand for alternative treatments (sustainable and ecofriendly) for both waste management and environmental accidents resulting from the petrochemical industry. Bioremediation is a viable alternative to remove contaminants, since biological treatments are cheaper than chemical and physical treatments, and occasionally result in complete mineralization (Van Hamme et al., 2003; Cappello et al., 2019). Bioremediation techniques for the recovery of polluted environments, such as autochthonous bioaugmentation, use only organisms that are indigenous to the community (Hassanshahian et al., 2014; Radwan et al., 2019).

Microbial consortia are preferred for the bioremediation of contaminants due to the presence of a large number of diverse metabolic functionalities (McGenity et al., 2012). The consortia can be constructed in defined or undefined ways (Alvarez and Polti, 2014). A defined consortium is an association of previously known microorganisms with a specific function, such as biodegradation (Smith et al., 2013; Alvarez and Polti, 2014). In contrast, undefined consortia result from enrichment procedures of environmental samples, including different organisms that may already be known (Sugiura et al., 1997; Hosokawa et al., 2009; Escalante et al., 2015; Santisi et al., 2015). While creating indefinite consortia, the samples are first grown in “rich or generic medium” with different carbon sources, and then submitted to a selection phase in minimal medium (Sugiura et al., 1997; Budzinski et al., 1998). Therefore, the undefined consortium approach may favor the selection of several organisms directly or indirectly involved in the biotechnological functions of interest, such as hydrocarbon degradation and production of biosurfactants (Venkateswaran, 1991).

Biosurfactants are amphipathic molecules that can be classified in different categories, one of which is by their molecular weight (Karlapudi et al., 2018). Low molecular weight biosurfactants are usually glycolipids (as rhamnolipids) or lipopeptides (as surfactin), while high molecular weight biosurfactants include amphipathic polysaccharides, lipopolysaccharides, proteins, and lipoproteins (Karlapudi et al., 2018; Nurfarahin et al., 2018). Most biosurfactants are synthesized by non-ribosomal pathways, however, the mechanisms that control their synthesis and production are poorly understood, which demands investigation (Nurfarahin et al., 2018). The recent discovery of a ribosomal protein with surfactant properties, named MBSP1, emphasizes the advantage of furthering the knowledge about biosurfactant biosynthesis (Araújo et al., 2020). Although various studies have described microbial consortia capable of bioremediation petrochemical waste through biosurfactant production, there are many questions about the role and production of biosurfactants for bioremediation (Ławniczak et al., 2013; Patowary et al., 2016; Lee et al., 2018).

In the current work, we used metagenomics to compare undefined microbial consortia, obtained by two different cultivation approaches using oil as a carbon source. We aimed to identify the factors that favor the enrichment of species, genes, and metabolic pathways related to petroleum degradation and biosurfactants production for biotechnological applications.

## Materials and Methods

### Obtaining Microbial Consortia

The PW sample used in this work was kindly provided by Petrogal Brasil S/A from onshore oil reservoir located in Aracajú, Sergipe, Brazil (Latitude: −10.9111099; Longitude: −37.0716705). Based on protocols adapted from Wu et al. (2013) and Guerra et al. (2018), the consortia were obtained in Erlenmeyer flasks containing 50 mL of the PW sample and culture medium in a 1:1 (v/v) of volume proportion, and were incubated at 30°C at 180 rpm for 7 days (1 cycle). After the first incubation cycle, 5% (v/v) of the culture was transferred to a fresh culture medium containing 1% (v/v) sterile petroleum by autoclaving at 135°C for 30 min. Incubation was performed under the same conditions and repeated twice, totaling three cycles. At the end of the third cycle, an aliquot (1% v/v) was transferred to Bushnell-Haas (BH) culture medium (g/L: 0.2 MgSO_4_, 0.02 CaCl_2_, 1 KH_2_PO_4_, 1 K_2_HPO_4_, 1 (NH_4_)_2_SO_4_, 0.05 FeCl_3_, pH 7) containing 1% (v/v) petroleum. Similar to the first three cycles described above, this phase was also repeated three times under the same conditions. The culture medium differentiated the consortia. The yeast extract peptone dextrose (YPD) consortium was obtained from the enrichment of the PW sample culture in the presence of the carbon-rich Peptone Dextrose Yeast Extract medium (g/L: 10 yeast extract, 20 peptone, 20 glucose) during the first three cycles and then transferred in the last three cycles for the selection phase with BH medium. The BH consortium was cultured for 6 weeks only with BH medium. The consortia were stored in a 1:1 (v/v) ratio of 100% glycerol (Synth^®^, Diadema, SP, BR) at −80°C for subsequent experiments. The petroleum used as a carbon source was sterilized in an autoclave for 3 cycles of 30 min.

### Growth Curves

Microbial growth was measured by optical density (OD_600nm_) in a spectrophotometer Global Analyzer, (Global Trade Technology, Monte Alto, SP, BRA) and performed in 250 mL Erlenmeyer flasks containing 50 mL of BH medium with 1% petroleum (v/v) as the sole carbon source, in triplicates. The initial OD_600nm_ was adjusted to 0.1. The consortia were grown over 7 days in the same conditions of incubation (30°C and stirring at 180 rpm) and verified every 24 h. All experiments with the consortia were standardized to be performed after 72 h of growth, which corresponds to the stationary growth phase of these consortia.

### DNA Extraction and Sequencing

DNA extraction of the consortia was performed during the stationary phase of the consortia by the commercial UltraClean Microbial DNA Isolation Kit (MoBio^®^, Carlsbad, CA, United States). For the DNA extraction of microorganisms present in crude PW, 5 L of sample were vacuum filtered in a 0.22 μm filter, and the membrane containing the retained microorganisms was cut into small pieces and used for DNA extraction by the commercial UltraClean Microbial DNA Isolation Kit (MoBio^®^, Carlsbad, CA, United States). The quality and quantity of DNA from all extractions (consortia and crude sample) were estimated using the Qubit 2.0 fluorometer (Thermo Fisher Scientific, Waltham, MA, United States).

All sequencings were performed according to the manufacturer’s instruction, suitable for the Ion Personal Genome Machine (PGM) platform (Thermo Fisher Scientific, Waltham, MA, United States). For shotgun metagenomic sequencing, 400 bp-fragment libraries were obtained using the Ion Xpress Plus Fragment library kit (Thermo Fisher Scientific, Waltham, MA, United States). The size selection of the library was performed by E-Gel SizeSelect 2% agarose (Invitrogen, United States) and purification steps using magnetic beads on Agencourt^®^ XP Kit (Beckman Coulter, United States), always following the manufacturer’s protocols. For 16S rDNA sequencing of the PW sample, 16S rRNA gene regions were amplified with 16S Ion Metagenomics Kit^TM^ (Thermo Fisher Scientific, Waltham, MA, United States). For each sample, two PCR reactions were carried out using primer sets V2-4-8 or V3-6,7-9. Ion Plus Fragment Library Kit^TM^ was used to obtain the DNA library with the adapter-ligated and nick-repaired DNA. In both sequencing methods, the Ion Xpress Barcode Adapters 1-32 kit^TM^ (Thermo Fisher Scientific, Waltham, MA, United States) was used to identify each sample. Subsequently, for both libraries (fragment and amplicon), emulsions were independently created and enriched using an Ion OneTouch^TM^ 2 System (Thermo Fisher Scientific, Waltham, MA, United States) and Ion PGM^TM^ Hi-Q View OT2 Kit (Thermo Fisher Scientific, Waltham, MA, United States). Sequencing was conducted according to Ion PGM^TM^ Hi-Q^TM^ View Sequencing Kit protocol on Ion 318 Chip v2 (Thermo Fisher Scientific, Waltham, MA, United States), following the manufacturer’s protocols. All runs were programmed to include 850 nucleotide flows.0

### Bioinformatics Analysis

Shotgun metagenomic raw sequences were uploaded to Metagenome Rapid Annotation using the subsystem Technology (MG-RAST)^1^ server (Keegan et al., 2016). Taxonomic analyses were performed using MG-RAST, with the default parameters, against the SILVA Databases (Quast et al., 2013). The biodegradation functional analyses of hydrocarbons and biosurfactant production were performed using the BLAST program (Gish et al., 1990) against the protein database of the Biosurfactant and Biodegradation Database (BioSurfDB) (Oliveira et al., 2015). For this analysis, Trimmomatic (Bolger et al., 2014) was used to remove quality index bases < 20, as well as fragments of sizes less than 30 bp. Read ends with high variation in nucleotide proportions were removed by the FastX trimmer, and duplicate reads were removed using FastQ/A collapser^2^. For the BioSurfDB alignment, the default BLAST parameters were used. All proteins identified in the metagenomes are available at http://www.biosurfdb.org/#/table/protein.

The program TrimGalore was used to pre-process amplicon sequences of 16S rRNA genes. Taxonomic determination was performed by QIIME (Caporaso et al., 2010), based on data generated by sequencing the V2–V9 region of the 16S rRNA. Chimera sequences were then excluded with USEARCH 6.1 (Edgar, 2018). Reads were clustered into OTUs at 97 % sequence identity using the UCLUST algorithm (Edgar, 2018). Representative sequences from each operational taxonomic unit (OTU) were mapped to the SILVA v132 database (Quast et al., 2013) to determine potential taxonomic identities.

We performed comparative bioinformatics analyses between the consortia obtained from this study, microbial consortia obtained from drill cutting samples (Guerra et al., 2018; Napp et al., 2018), and non-oil samples (Leinonen et al., 2010). From drill cutting samples, Napp et al. (2018) and Guerra et al. (2018) obtained consortia only with enriched culture medium. Both authors obtained consortia from the Luria Broth culture medium, named LBE consortium in Napp et al. (2018) and BHLBL consortium in Guerra et al. (2018). Furthermore, Napp et al. (2018) obtained a consortium in the Potato Dextrose medium (PDE consortium) and Guerra et al. (2018) from the YPD medium (BHYPDL consortium). Two consortia obtained without oil as carbon source were used as control. The metagenomic sequences of the phototrophic (SRX375283) and cellulose degradation (SRX474425) consortia were obtained from the public database Sequence Read Archive (SRA)^3^ (Leinonen et al., 2010) and used for comparison, being control 1 and 2, respectively. The complete description of these consortia is available in the Supplementary Table S1. The consortia were aligned using the BLAST program (Gish et al., 1990) with the protein database BioSurfDB, and the cluster analysis was performed by studio R (version X). All these consortia were deposited in the online database BioSurfDB.

### Emulsification Index (E_24%_) Determination

The production of emulsifiers was assessed during the stationary phase. Then, emulsification activity was measured according to Cooper and Goldenberg (1987) with minor adaptations. In brief, 2 mL of different hydrocarbons (kerosene, naphthalene, decane, hexadecane, dichloromethane, and hexacosane) were added to 2 mL of cell-free supernatant from cultures grown in BH medium with petroleum as the only carbon source. The mixture was agitated in vortex at high speed (3,000 rpm) for 2 min and then left to stand for 24 hat room temperature. Emulsification activity was calculated as the height of the emulsion layer divided by the total height and multiplied by 100. As naphthalene and hexacosane were diluted in dichloromethane, the emulsification index (E24%) dichloromethane was subtracted from naphthalene and hexacosane indexes. Culture medium BH and SDS 1% were used as negative and positive controls, respectively.

### Interfacial Tension

The evaluation of interfacial tension was performed according to Araújo et al. (2020) in a tensiometer, model DVT50 (Krüss, Hamburg, DEU) using the Rising Drop method, as recommended by the manufacturer. The assay was performed between two phases, the interfacial force between the liquid containing surfactant (bulk phase), and the oil droplet formed in dispense phase is evaluated. Culture supernatant of BH medium was the bulk phase, and petroleum was the other phase. The consortia were cultivated for 5 days until the late stationary phase. Cell-free supernatant (15 mL) was obtained by centrifugation at 5,583 × *g* for 15 min. SDS (1 %) and distilled water were used in the bulk phase as positive and negative controls, respectively.

### Cell Hydrophobicity

The hydrophobicity of cells was evaluated as described by Rosenberg (1984) with modifications. The consortia were cultivated 5 days until the late stationary phase. The pellet was obtained by centrifugation at 5,583 × *g* for 15 min and was then washed and resuspended in PUM buffer (g/L) (22.2 K_2_HPO_4_3H_2_O, 7.26 KH_2_PO_4_, 1.8 urea, 0.2 MgSO_4_7H_2_O, pH 7). The culture was diluted in PUM buffer to OD_600nm_ 0.5, and 3 mL was added to a penicillin flask with 500 μL of chloroform. The flask was homogenized in a vortex at high speed (3,000 rpm) for 2 min and incubated at room temperature for 1 h in rest. The triplicates of aqueous phase were read at OD_600nm_, and the hydrophobicity was estimated based on the following equation 01: Cell hydrophobicity (%) = (OD before−OD after/OD before) × 10.

### Biodegradation Percentage Determination

The assay was performed with a consortia pre-inoculum after a correction of the initial OD_600nm_ to 0.1 in Erlenmeyer flasks of 250 mL containing 50 mL of BH medium with 1% of petroleum as the sole source of carbon and cultivated for 24 days at 30°C with stirring at 180 rpm. After the incubation period, the biodegradation percentage of both aliphatic hydrocarbons and polycyclic aromatic hydrocarbons (PAHs) by microbiological consortia from oil reservoirs were determined by a gas chromatography-flame ionization detector (GC-FID) on a Clarus^®^ 600 Chromatograph Adapter (PerkinElmer, Inc., Waltham, MA, United States). The samples were subjected to a liquid-liquid extraction process and to preparative liquid chromatography to clean up the aliphatic and PAH fractions for carrying out quantitative analysis by GC-FID based on (Pugazhendi et al., 2017; Napp et al., 2018; Pereira et al., 2019). The identification of aliphatic hydrocarbons and PAH constituents was performed according to Dörr de Quadros et al. (2016) and Napp et al. (2018). Quantitative analyses were performed using the modified external standardization method according to Pereira et al. (2019) and Araújo et al. (2020). Biodegradation percentage was calculated based on the following equation: 02. B_*p*_ = [(C_*i*_−C_*f*_)/C_*i*_] × 100, where *B*_*p*_, *C*_*i*_, and *C*_*f*_ are the biodegradation percentages at the end of the incubation time, the amount of contaminant at the start of incubation, and the amount of contaminant at the end of incubation, respectively (Araújo et al., 2020).

### Statistical Analysis

Comparative taxonomic and functional analyses were performed using statistical analyses of taxonomic and functional profiles (STAMP) (Parks et al., 2014). The significant differences between the relative proportions were calculated using the two-sided Fisher’s exact test with the Newcombe–Wilson confidence interval method, using Storey’s false discovery rate (FDR) and the Benjamin–Hochberg FDR for correction. Results with *q* < 0.05 (corrected *p*-value) were considered significant; unclassified reads were removed from the analysis. For all other experimental assays, a two-way ANOVA, *t*-test, or two-sided Fisher’s exact test was used when appropriate and considered significant at *p* < 0.05.

## Results

### Consortia Growth Behavior

**FIGURE 1:**
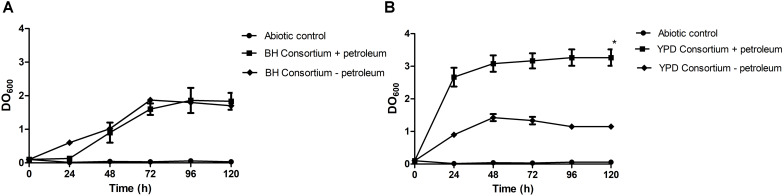
Consortia growth curves in mineral medium with 1% (v/v) sterile petroleum. **(A)** Growth curve of the BH consortium + petroleum, compared to the negative controls, one containing only mineral medium and autoclaved oil (Abiotic control), and the other containing BH consortium in mineral medium without oil (BH consortium – petroleum). **(B)** Growth curve of the YPD consortium + petroleum, compared to the negative controls (Abiotic control and YPD consortium – petroleum). The statistical significance was analyzed by a two-way ANOVA, and *p* < 0.05 was considered significant (*p < 0.05).

In this work, enrichment and selection approaches were used to obtain hydrocarbon-degrading microbial consortia, derived from a Brazilian reservoir PW sample. The first approach consisted of using a rich medium (YPD) in the enrichment step, previously to the selection step with mineral medium (BH), while in the second approach, both steps were performed only in BH medium, containing oil as the only carbon source. In order to assess the hydrocarbon assimilation potential, microbial growth behavior of undefined consortia with or without the addition of petroleum was evaluated. The consortia presented different growth dynamics until the stationary phase in the presence or absence of oil. The stationary phase of the YPD consortium was reached after 24 h, while the population density of the BH consortium stabilized only after 72 h of growth. Therefore, subsequent experiments were carried out after 3 days. It was observed that the consortia also grew in the absence of petroleum. The BH consortium presented similar growth regardless of the presence or absence of oil, and the YPD consortium showed a significant difference between growth in the presence of oil compared to the medium without oil (Figure 1).

### Metagenomic Diversity

**FIGURE 2:**
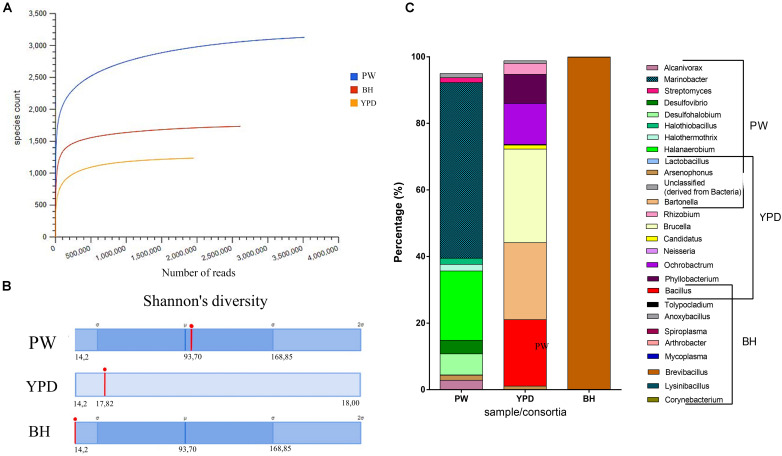
Taxonomic diversity of the samples. **(A)** Rarefaction curve of samples sequenced by the shotgun metagenomics analysis method and analyzed using MG-RAST. Blue curve = production water (PW); red curve = BH consortium; and yellow curve = YPD consortium. **(B)** Comparison of the Shannon’s diversity indices (red line) between the three sequenced samples. Automatic annotation performed by the MG-RAST pipeline. **(C)** Most abundant genera identified in the production water (PW), YPD consortium, and BH consortium.

In order to access the taxonomic and metabolic diversity profiles of the PW crude sample and consortia obtained, metagenomic analyses were carried out. General information regarding each sample submitted for shotgun metagenome sequencing is available in the Supplementary Table S2. Rarefaction curves were obtained, and the plateau was reached for all metagenomic samples, meaning that sequencing was deep enough to cover the diversity of species in each community (Figure 2A). Both consortia showed a reduction in diversity in relation to the environmental PW sample (Figure 2B). Although the BH consortium presented a higher number of species (Figure 2A), these predominantly (over 90%) belonged to the genus *Brevibacillus* (Figure 2C), in accordance with the low Shannon index (Figure 2B). On the other hand, the YPD consortium presented several genera in equitable proportions (Figure 2C). In addition, the BH consortium had the larger proportion of annotated proteins compared to YPD consortium (Supplementary Table S2).

### Taxonomic Abundance

The microbial community of the PW sample was analyzed by sequencing of the 16S rDNA gene and shotgun metagenome (Table 1). The most abundant genera in the PW metagenome were *Marinobacter*, *Halanaerobium*, *Desulfohalobium*, *Desulfovibrio*, and *Alcanivorax* (Table 1), but these predominant genera were not enriched in the consortia. Furthermore, the most enriched genera in the consortia were present in low abundance in the PW sample. Therefore, consortium cultivation favored the growth of rare genera, since non-abundant genera in the PW sample were found to be among the most prevalent in cultivation.

**TABLE 1:** Relative abundance of genera in production water by shotgun metagenomic and rDNA 16S sequencing methods.

**Abundance (%)**			

**Production water (Shotgun sequencing)**		**Production water (16S rDNA sequencing)**	
*Marinobacter*	52.74	*Halanaerobium*	27.85
*Halanaerobium*	20.83	*Marinobacter*	11.82
*Desulfohalobium*	6.34	*Alcanivorax*	9.38
*Desulfovibrio*	4.07	*Desulfovibrio*	3.77
*Alcanivorax*	2.84	*Brevibacillus*	0.31
*Halothermothrix*	1.98	*Ochrobactrum*	0.30
*Halothiobacillus*	1.85	*Bacillus*	0.27
*Arsenophonus*	1.50	*Desulfohalobium*	0.21
*Streptomyces*	1.45	*Rhizobium*	0.06
unclassified (derived from Bacteria)	1.26	*Phyllobacterium*	0.01
*Bartonella*	0.08	*Paenibacillus*	0.01

**FIGURE 3:**
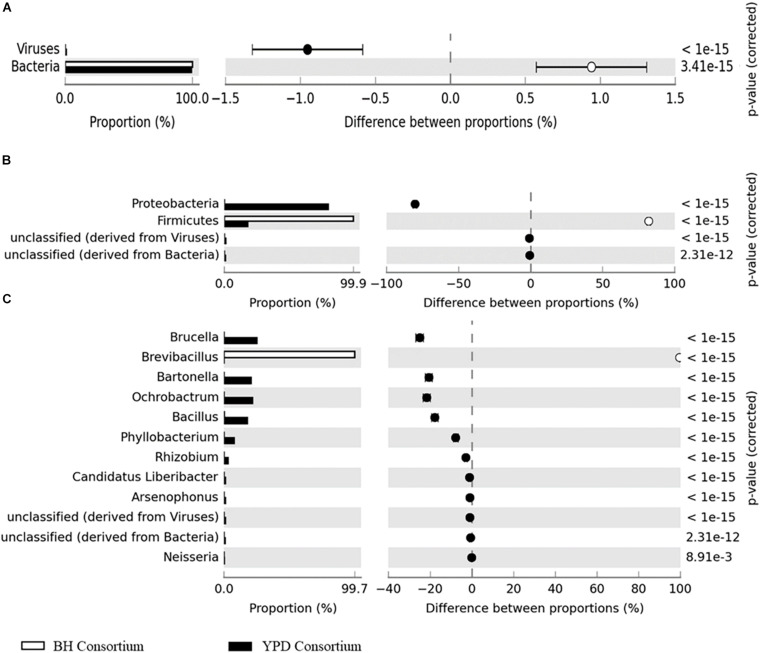
Differences in taxonomic profiles between consortia. **(A)** Domain; **(B)** Phylum; and **(C)** Genus levels. *q* < 0.05 was considered statistically significant, using the two-sided Fisher’s exact test.

Significant differences (*q* < 0.05) at the domain, phyla, and genera level between consortia were presented in Figure 3. There is a predominance of the Bacteria domain in both consortia, similarly to that observed for the crude sample. However, at the phylum level there is a predominance of Proteobacteria in the YPD consortium, while Firmicutes predominated in the BH consortium. As described above, the BH consortium was predominantly composed of representatives of the genus *Brevibacillus* (99,73%). Low abundance genera (frequency < 1%), such as *Bacillus*, *Spiroplasma*, *Lactobacillus*, *Arthrobacter*, and *Corynebacterium*, were also detected in the BH consortium. In the YPD consortium, *Brucella* was the most abundant genus (28,11%), but there was no predominance over the other bacterial genera, such as *Bacillus* (28,11%), *Bartonella* (23,14%), *Bacillus* (19,94%), and *Ochrobactrum* (12,34%) (Table 2). In addition, YPD cultivation favored the enrichment of genera classified as non-cultivable or unclassified genera derived from Bacteria.

**TABLE 2:** Frequency of the 10 most abundant genera in the consortia identified by shotgun metagenomic sequencing.

**Abundance (%)**			

**YPD consortium**		**BH consortium**	
*Brucella*	28.11	*Brevibacillus*	99.73
*Bartonella*	23.14	*Bacillus*	0.11
*Bacillus*	19.94	*Spiroplasma*	0.07
*Ochrobactrum*	12.34	*Lactobacillus*	0.02
*Phyllobacterium*	8.72	*Anoxybacillus*	0.01
*Rhizobium*	3.29	*Arthrobacter*	0.01
*Candidatus Liberibacter*	1.21	*Corynebacterium*	0.01
*Arsenophonus*	1.07	*Lysinibacillus*	0.01
*unclassified (derived from Viruses)*	1.07	*Mycoplasma*	0.01
*unclassified (derived from Bacteria)*	0.79	*Tolypocladium*	0.01

### Comparative Functional Profiles

**FIGURE 4:**
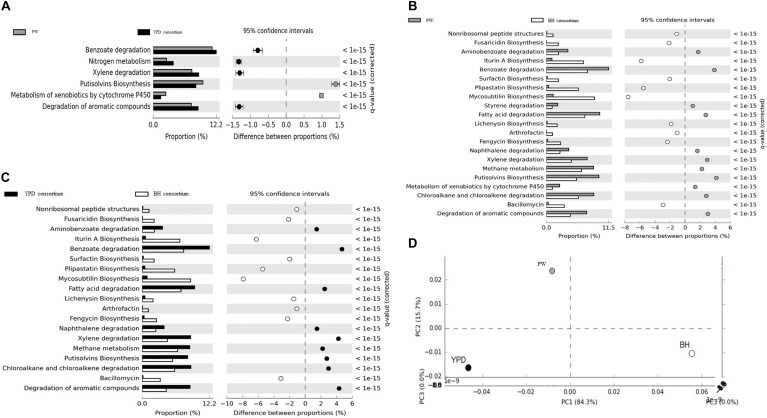
Functional annotation of shotgun metagenome sequencing performed using the BioSurfDB database. **(A)** Statistical differences in metabolic pathways between YPD consortia and PW. **(B)** Statistical differences in metabolic pathways between the consortia BH and PW. **(C)** Functional comparison between the consortia BH and YPD. **(D)** Principal component analysis between BH and YPD consortia and PW. The graph shows the statistical differences between functional profiles observed in the metagenomes. *q* < 0.05 was considered statistically significant, using the two-sided Fisher’s exact test.

In order to identify genes and metabolic pathways related to hydrocarbon degradation and production of biosurfactants, the metagenomes obtained were aligned against the BioSurfDB database. The results suggested that YPD medium favored the growth of a consortium enriched in hydrocarbon degradation genes, compared to the BH consortium. The main pathways enriched are involved in the degradation of aromatic compounds, nitrogen metabolism, methane, and metabolism of xenobiotic compounds by cytochrome P450 (Figures 4A,C). In contrast, biosurfactant biosynthesis pathways, such as iturin A, liquenisin, surfactin, and plipastatin, were enriched in the BH consortium, except for the Putisolvin biosurfactant, which was more abundant in YPD consortium (Figures 4B,C). Although both consortia were obtained under the same functional selection in BH with oil, the functional profiles analyzed by principal component analysis (PCA) showed that the consortia are not metabolically similar (Figure 4D). Thus, the YPD culture media exerted different selective pressures on the YPD consortium.

**FIGURE 5:**
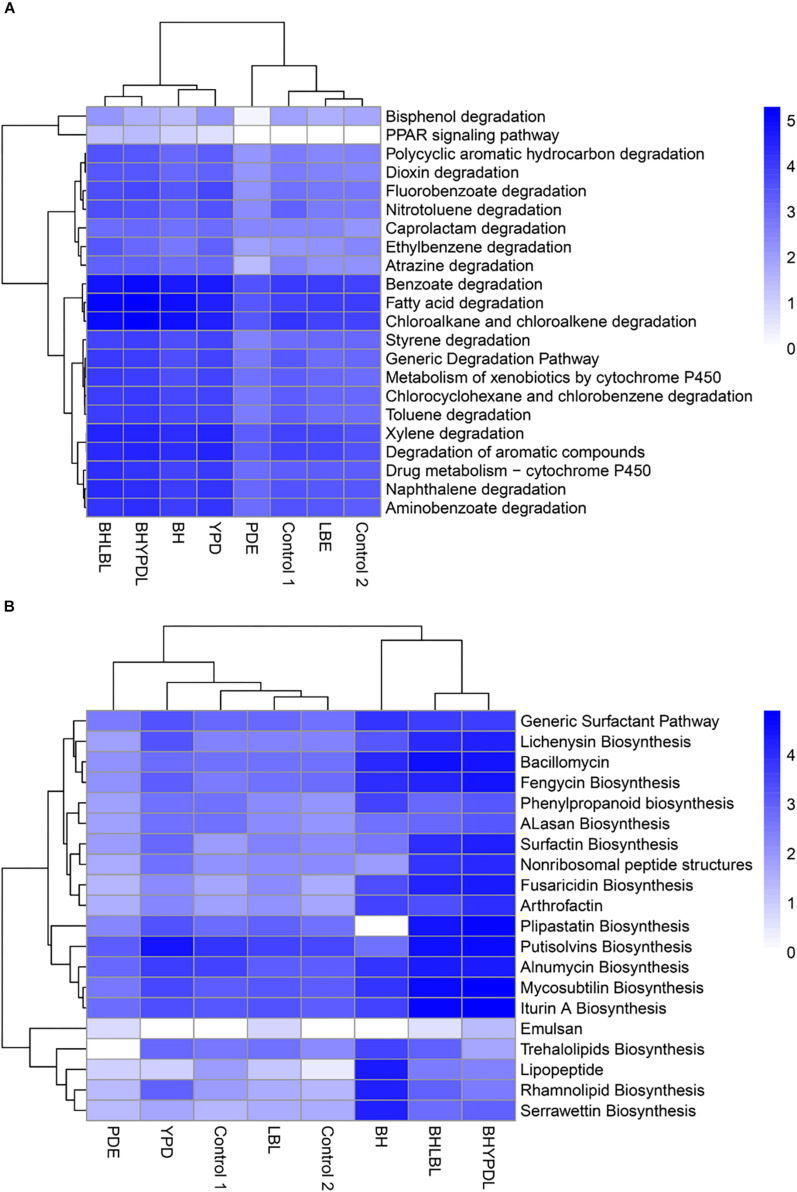
Cluster analysis of biodegradation and biosurfactant biosynthesis pathways in the consortia. The influence of the consortia obtainment medium on the abundance of biodegradation and biosurfactant genes was analyzed. **(A)** Clustering of genes involved in hydrocarbon biodegradation pathways. **(B)** Clustering of genes involved in biosurfactant biosynthesis pathways. The clustering was based on the abundance of reads annotated in each pathway. Heatmap colors are generated with log_10_ among the individual samples. A heatmap was generated using the heatmap package in the statistical program R.

To identify the set of genes that specifically responded to selection in oil as the sole carbon source, we performed a comparative analysis between the consortia we obtained in this work and other consortia obtained in previous works of our group, Napp et al. (2018), and Guerra et al. (2018), as well as a control without oil from the databank SRA (SRX375283 and SRX474425). The heatmap (Figure 5A) shows a vertical cluster formed by the metagenomes of BHYPDL, BHLBL, BH, and YPD consortia, which all underwent the common selection step in BH medium. This cluster presented an enrichment of pathways related to degradation of more complex hydrocarbons, and the Peroxisome proliferator-activated receptor (PPAR) signaling pathway was exclusive for this cluster. In fact, this pathway (PPAR) is finder in a eukaryotic cell. The alkane hydroxylase (*AlkB* gene) is the only bacterial enzyme associated with this pathway due to a similar activity to that of the non-heme integral-membrane acyl coenzyme A (CoA) desaturases and acyl lipid desaturases (Shanklin and Whittle, 2003). In addition, there is a horizontal cluster formed by degradation pathways of simple hydrocarbons, such as fatty acids, benzoate, and chloroalkanes, with no differences between consortia grown with or without oil, regardless of the culture medium.

The metagenomes of the BH, BHLBL, and BHYPDL consortia also formed a cluster with regard to the abundance of genes involved in biosurfactant biosynthesis (Figure 5B). The step through BH as minimal medium, proved to be more effective in the selection of biosurfactant biosynthesis pathways, since the consortium obtained in rich media presented a reduced abundance of these genes. The lipopeptide, serrawettin, trehalolipid, and rhamnolipid biosynthesis pathways were mainly enriched in the consortium that were selected only in BH medium with hydrophobic substrate, such as oil (Figure 5B).

### Production of Biosurfactants

**FIGURE 6:**
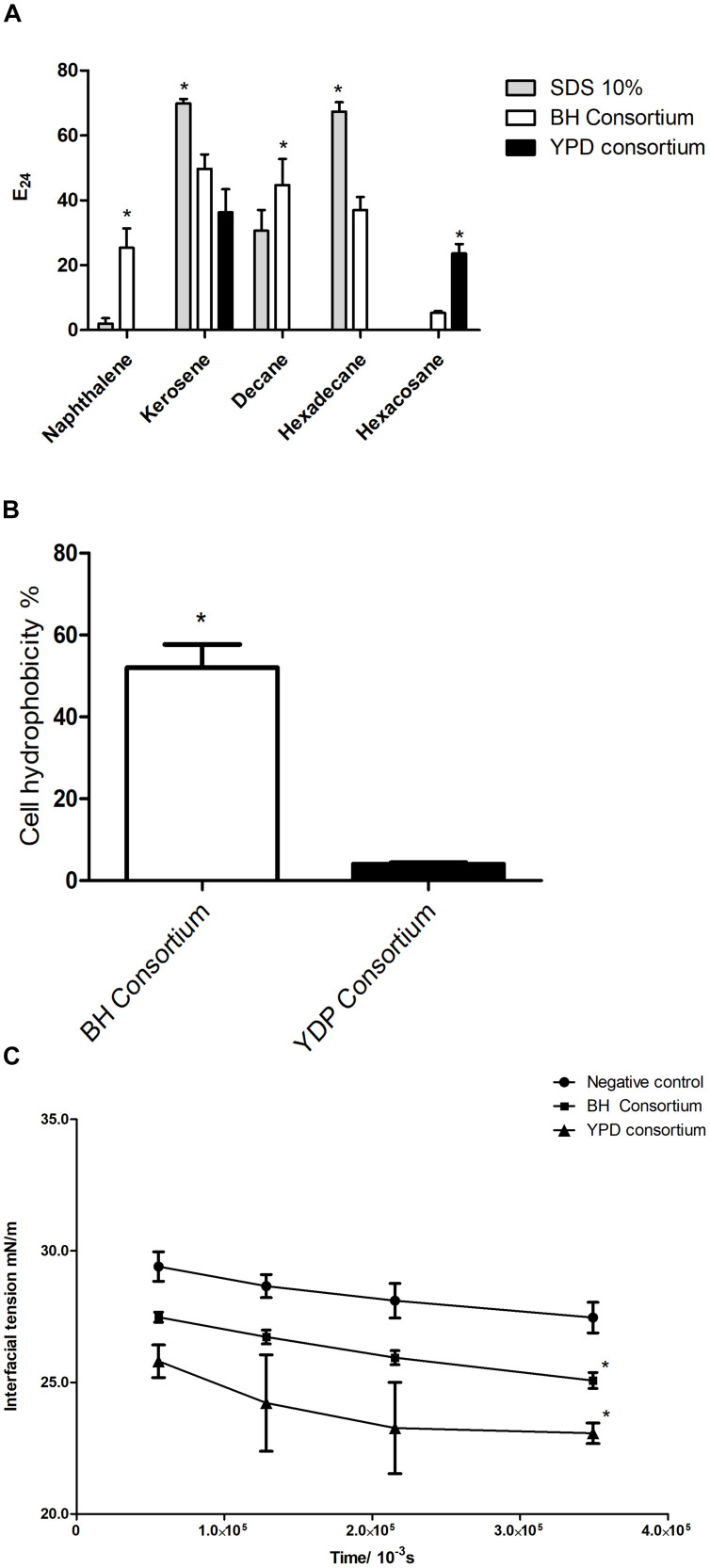
Production of biosurfactants by consortia. **(A)** Emulsification indexes (E24%) for BH and YPD consortia in different hydrocarbons (naphthalene, kerosene, decane, hexadecane, and hexacosane). The statistical significance was analyzed by a two-way ANOVA (*p < 0.05). **(B)** Cell hydrophobicity in YPD and BH consortia. These were analyzed using a *t*-test (*p < 0.05). **(C)** Evaluation of the interfacial tension in relation to the time of release of the oil droplet in contact with the supernatant of the BH and YPD consortia. This result was analyzed by a two-way ANOVA (*p < 0.05 compared to the control).

To assess the presence of extracellular surfactant produced by the consortia, cell-free supernatant was analyzed for the formation of stable emulsion and the ability to reduce interfacial tension. The YPD consortium presented emulsification only in kerosene and hexacosane, whereas the BH consortium showed a significant emulsification capacity in all hydrocarbons tested and was the only one to emulsify an aromatic hydrocarbon (naphthalene) (Figure 6A). This result is in line with the greater abundance of biosurfactant genes observed in the BH consortium (Figure 5B).

Some biosurfactants are found adherent to cell membranes giving high hydrophobicity to the cell membrane (Ramkrishna, 2010; Soberón-Chávez and Maier, 2011). Therefore, cellular hydrophobicity was measured in consortia. The BH consortium presented a microbial community that was significantly more hydrophobic than the YPD consortium, 52 and 4%, respectively (Figure 6B). In contrast, the YPD consortium showed greater capacity to reduce interfacial tension when compared to the BH consortium (Figure 6C).

### Biodegradation of Aliphatic and Polycyclic Aromatics Hydrocarbons

**FIGURE 7:**
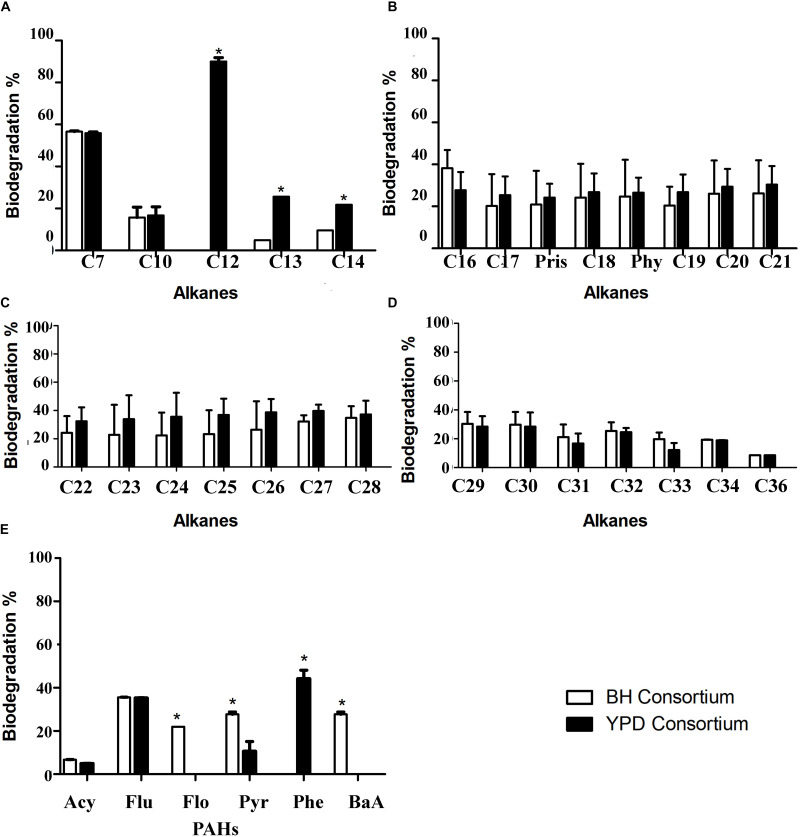
Hydrocarbon biodegradation profiles of the BH and YPD consortia. **(A)** Comparison of biodegradation of short aliphatic hydrocarbons (from 7C to 14C) in percentages; **(B)** Biodegradation of aliphatic hydrocarbons (from 15C to 21C); **(C)** Biodegradation of long aliphatic hydrocarbons (from 22C to 28C); **(D)** Biodegradation of long aliphatic hydrocarbons (from 29C to 36C); and **(E)** Biodegradation of total polycyclic aromatic hydrocarbons (PAHs), in percentage, compared to the negative control only with mineral medium and petroleum analyzed by chromatography. All data were analyzed using two-way ANOVA (*p < 0.05).

The biodegradation of aliphatic and PAHs by the consortia was evaluated using GC-FID. The comparison of aliphatic hydrocarbon biodegradation in general showed no statistical difference between consortia (Figures 7A–D and Supplementary Figure S1). Regarding the alkane degradation, statistical differences were only observed in C12 to C14 degradation, in which YPD showed a significant increase compared to BH consortium. The biodegradation of PAHs, acetylene (Acy), fluorene (Flu), pyrene (Pyr), also occurs in both consortia, and there is no statistical difference for the first two PAHs. However, exclusive biodegradation of phenanthrene (Phe) occurs in the YPD consortium, whereas biodegradation of fluoranthene (Flo) and benzo[a]anthracene (BaA) occurs in the BH consortium (Figure 7E). Therefore, there was more biodegradation of different types of PAHs in the consortium cultivated only in mineral BH medium.

## Discussion

In the current work, two consortia from the same PW sample were obtained using different methods in order to test the medium influence on microbial diversity, hydrocarbon biodegradation, and biosurfactant production genes. Our data demonstrated that the YPD consortium was taxonomically more diverse and enriched in hydrocarbon degradation genes, while the BH consortium showed *Brevibacillus* predominance and enrichment in several biosurfactants production genes. These findings might improve the production efficiency of low molecular weight biosurfactants from hydrophobic substrates such as oil. Few studies focus on the interactions between hydrocarbons biodegradation and biosurfactant families simultaneously. Some works described that bacteria compete for hydrocarbon substrates through biosurfactants production, which have antimicrobial activity and improve their ability to biodegrade hydrocarbons (Cray et al., 2013; Ndlovu et al., 2017). However, there are studies that demonstrate that microbial consortia can present higher rates of hydrocarbon degradation and biosurfactants production than isolated species (Cerqueira et al., 2011; Araújo et al., 2020).

The presence of biosurfactant favors a better efficiency of hydrocarbon degradation in microbial consortia with a slow growth rate. In general, slow-degrading consortia (such as BH consortium) perform hydrocarbon absorption from the aqueous phase through their solubilization using biosurfactants (Ławniczak et al., 2013). In addition, BH consortium growth was observed even in the absence of oil, which suggests the occurrence of CO_2_ sequestering species. This was corroborated by the data obtained from the sequencing, which showed the presence of reads annotated as carbonic anhydrase attributed to the genus *Brevibacillus*. This enzyme catalyzes the interconversion between carbon dioxide and bicarbonate, being used by bacteria to capture CO_2_ (Boone et al., 2013; Supuran and Capasso, 2017). The CO_2_ sequestration capacity has been described for the *Bacillus* sp. SS105. The authors observed activity of carbonic anhydrase and ribulose-1,5-bisphosphate carboxylase/oxygenase (RuBisCO) associated with CO_2_ sequestration and increased production of biosurfactants, suggesting that the use of this strain for CO_2_ fixation as a strategy to mitigate CO_2_ emissions (Maheshwari et al., 2017, 2018). Considering our results, it is plausible to propose that *Brevibacillus* has the same capacity, which should be investigated in the future.

In contrast to slow-growing bacteria, fast-growing consortia, such as YPD, tend to form biofilms at the interfacial frontier, which suggests that direct capture mechanisms are prevalent. In this case, biosurfactants may be synthesized as secondary metabolites after degradation of the oil-water interface (Ławniczak et al., 2013). However, in this work, the analysis of biosurfactants was made when both consortia were in the stationary phase, i.e., during the highest production of biosurfactants (Lin, 1996; Fontes et al., 2012; Rufino et al., 2014).

Low-abundant genera in the PW were enriched in both consortia. This proportion may be due to the PW anaerobic conditions in the oil reservoir (Wang et al., 2011), contrasting with the culture aerobic conditions used in this work. Although anaerobic taxa are prevalent, there is a great abundance of aerobic bacteria in oil reservoirs (An et al., 2013). We observed the predominance of the *Brevibacillus* genus in BH consortia. This genus has been reported in oil reservoir samples and associated to oil degradation and biosurfactant production (Mnif et al., 2011; She et al., 2014), which has allowed for its application in biotechnological approaches for microbial enhanced oil recovery (MEOR) (Hou et al., 2005, 2008, 2011; She et al., 2012; Purwasena et al., 2018). However, the genes and metabolic pathways involved in these processes are poorly understood.

The comparison between consortia from this work and publicly available consortia showed that the presence of oil during the microbial growth imposed weak selective pressure for the enrichment of simple hydrocarbon degradation pathways, such as fatty acid, aliphatic alkane, and monoaromatic hydrocarbons, since all consortia showed a similar abundance of genes involved in these degradation pathways. Thus, the presence of oil in the environment does not seem to be a limiting factor for the enrichment of simple hydrocarbon degradation genes, since these genes are part of the basal lipid metabolism (Yao and Rock, 2017). An exception was the *AlkB* gene (alkane hydroxylase), associated with both the fatty acid and the PPAR pathways, which was enriched only in consortia that had undergone the selection stage in BH medium. AlkB is an integral-membrane di-iron enzyme that plays a pivotal role in aerobic degradation of alkanes and is widely distributed in bacteria (Nie et al., 2014).

Although the YPD consortium presented a greater predominance of genes related to the hydrocarbon degradation, few significant differences in the aliphatic hydrocarbon degradation were observed when compared to BH consortium. However, the BH consortium degraded more types of aromatic hydrocarbons than the YPD consortium, suggesting a synergistic effect caused by biosurfactants (Kim et al., 2009; Alvarez and Polti, 2014; Perera et al., 2019). Biosurfactants allow hydrophobic molecule solubilization, increasing their bioavailability (Nitschke and Costa, 2007). Different biosurfactants can solubilize more hydrocarbons types, promoting the assimilation of hydrocarbons onto the cell membrane by changes of cell hydrophobicity and bioavailability (Phale et al., 1995; Prabhu and Phale, 2003; Andreoni et al., 2004; Kim et al., 2009). In addition, it has been described that Firmicutes, abundant in the BH consortium, are an indicator of the later stages of hydrocarbon degradation when more recalcitrant compounds, such as PAHs, are present (Beazley et al., 2012). As described by Dasari et al. (2014), biosurfactants are useful for PAH biodegradation, which is in line with our results, since we observed PAH degradation and occurrence of emulsifying activity in the BH consortium. Altogether, these results suggest that the BH consortium, although less diverse and presenting less reads related to hydrocarbon degradation, has a community capable of degrading oil due to the production of biosurfactants.

The YPD and BH consortia also differ in their ability to reduce interfacial tension and emulsify different hydrocarbons. The YPD consortium was more efficient in reducing interfacial tension. This consortium stands out for the higher occurrence of genes related to Putisolvin biosynthesis. Low molecular weight biosurfactants, such as the lipopeptide Putisolvin, are more effective in reducing interfacial tension (Al-Araji et al., 2007; Satpute et al., 2010; Uzoigwe et al., 2015; Jahan et al., 2020). This biosurfactant has been described in *Pseudomonas* species, with no report of its production by other genera (Dubern et al., 2005, 2006; Dubern and Bloemberg, 2006). In this work, *Pseudomonas* is not among the most abundant genera in the YPD consortium, suggesting that other genera may be responsible for the production of Putisolvin. In fact, the BLAST analysis indicated occurrence of Putisolvin genes in *Bacillus*, which is one of the most abundant genera in the YPD consortium.

The BH consortium can emulsify more hydrocarbon types compared to the YPD consortium and shows enrichment of several biosurfactant classes. It has been proposed that high molecular weight biosurfactants and bioemulsifiers form better emulsions (Al-Araji et al., 2007; Satpute et al., 2010; Uzoigwe et al., 2015; Jahan et al., 2020). As an example, the cluster formed by BH, BHLBL, and BHYPDL consortia showed enrichment of some high molecular weight biosurfactants such as alasan, which is an anionic polysaccharide that possesses effective PAH emulsifying activity (Toren et al., 2002). Viramontes-Ramos et al. (2010) observed that strains grown in mineral medium supplemented with olive oil showed a higher rate of emulsification in kerosene, compared to strains grown in mineral medium supplemented with glucose. Together, the data indicate that cultivation in a minimal medium supplemented with oil is a more effective strategy for the selection of microorganisms producing biosurfactants with emulsifying activity.

The biosynthesis pathways for low molecular weight biosurfactants, such as lipopeptides, serrawettin, trehalolipid and rhamnolipids are also more prevalent in the BH consortium and may explain the high cell hydrophobicity in this consortium, since some biosurfactants can bind to the cell membrane and alter its hydrophobicity due to the presence of hydrocarbons (Rosenberg et al., 1988; Franzetti et al., 2008; Banat et al., 2010). Trehalolipid biosynthesis is induced by the presence of hydrophobic substrates, resulting in both membrane-bound as well as trehalolipids secreted to the extracellular medium promoting greater cellular hydrophobicity and hydrocarbon emulsification (Franzetti et al., 2010; Pacheco et al., 2010; Liu and Liu, 2011; White et al., 2013; Edosa et al., 2018). Lipopeptides and rhamnolipids are produced both in the presence of hydrophobic and hydrophilic substrates in mineral medium (Mukherjee and Das, 2005; Abdel-Mawgoud et al., 2011). By using oil as a carbon source, the production of rhamnolipids was 80% higher, compared to other hydrophobic substrates and almost 100% higher than with glucose (Mata-Sandoval’ et al., 2001). Rhamnolipids may be attached to the cell membrane allowing a more efficient hydrocarbon uptake by rendering the cell surface more hydrophobic (Al-Tahhan et al., 2000; Banat et al., 2010). Serrawettin, trehalolipid and rhamnolipid were not yet identified in *Brevibacillus*. However, the genes related to the production of these biosurfactants are more abundant in the BH consortium, suggesting that *Brevibacillus* species can produce these low molecular weight biosurfactant. The extraction and characterization of the biosurfactants produced by BH consortium will confirm this hypothesis.

In terms of yields, studies show that the production of biosurfactants and hydrocarbons biodegradation in consortia is greater than in culture of isolated bacteria (Jing et al., 2007; Mukred et al., 2008; Cerqueira et al., 2011). In contrast, Antoniou et al. (2015) using a defined consortia showed that the isolates produces more low-molecular weight biosurfactants, specially rhamnolipids and sophorolipids, than bacterial consortia. However, the latter produces a greater diversity of low-molecular weight biosurfactants, which may reflect the growth competition between microorganisms. Therefore, the success in the production of biosurfactants seems to depend on the interactions established between microorganisms present in the environment, such as synergism or competition, which needs to be further investigated.

Several studies have analyzed the influence of biosurfactants on bioremediation processes, mainly in terms of increased efficiency, which has been associated with better solubilization of pollutants, resulting in greater bioavailability and consequently higher rates of biodegradation (Makkar and Rockne, 2003; Araújo et al., 2020). In general, most of the works that enrich microorganisms for biosurfactant production use hydrophilic carbon sources. Simple sugar, starch, and plant sugar-based carbohydrates are the major carbon sources used as substrates (Nurfarahin et al., 2018). In this work, a consortium obtained only in mineral medium with oil as carbon source showed greater efficiency and competitiveness in the biodegradation of hydrocarbons through the production of biosurfactants.

## Conclusion

The microbial enrichment in minimal medium containing petroleum as carbon source was shown to be effective for the selection of microorganisms that produce biosurfactants. The BH consortium obtained proved to be efficient in hydrocarbon degradation, being superior to the YPD consortium in relation to the degradation of some PAHs. The selective pressure exerted by minimal medium containing oil led to the obtainment a consortium with low diversity in bacterial genera, constituted predominantly by representatives of the genus *Brevibacillus*, indicating that this is an efficient producer of different biosurfactant classes. Finally, our data indicate the biotechnological potential of consortia selected in a minimal medium containing oil, applicable for approaches as MEOR, CO_2_ capture, and bioremediation of waste and impacted areas.

## Data Availability Statement

The datasets presented in this study can be found in online repositories. The names of the repositories and accession numbers can be found at: http://www.mg-rast.org/, 4726099.3; http://www.mg-rast.org/, 4827355.3; http://www.mg-rast.org/, 4826970.3; http://www.mg-rast.org/, 4643476.3; http://www.mg-rast.org/, 4643480.3; http://www.mg-rast.org/, 4583777.3; http://www.mg-rast.org/, 4583773.3; https://www.ncbi.nlm.nih.gov/, SRX375283; https://www.ncbi.nlm.nih.gov/, SRX474425.

## Author Contributions

WA, JO, MF, SA, CM, RS-P, KS-B, AN, JP, and JF carried out the experiment, data analysis, and wrote the manuscript. MP, LP, MV, and LA-L were responsible for the conception, experimental design, and supervision of the project, as well as data analysis and wrote the manuscript. All authors read and approved the final version of the manuscript.

## Conflict of Interest

The authors declare that the research was conducted in the absence of any commercial or financial relationships that could be construed as a potential conflict of interest.

## Footnotes

1 http://metagenomics.anl.gov

2 http://hannonlab.cshl.edu/fastx_toolkit/

3 https://www.ncbi.nlm.nih.gov/sra/

## Abbreviations

Acy: acetylene
BaA: benzo[a]anthracene
BH: Bushnell-Haas
E_24%_: emulsification index
Flo: fluoranthene
Flu: fluorene
GC-FID: gas chromatography-flame ionization detector
MEOR: microbial enhanced oil recovery
OD_600nm_: optical density
PAHs: polycyclic aromatic hydrocarbons
PCA: principal component analysis
PGM: Personal Genome Machine
Phe: phenanthrene
PPAR: peroxisome proliferator-activated receptor
PW: production water
Pyr: pyrene
YPD: yeast extract peptone dextrose.
