# Supplementary material for: Microbial Culture in Minimal Medium With Oil Favors Enrichment of Biosurfactant Producing Genes
Source: Front Bioeng Biotechnol. 2020 Aug 11;8:962. doi: 10.3389/fbioe.2020.00962 (PMC7431673; doi:10.3389/fbioe.2020.00962)
Supplement: Supplementary file 1 [file Data_Sheet_1.docx]

# Supplementary material

Table S1 - Description of consortia deposited in databases

| Consortium name | Enrichment medium | Selection medium | Carbon source | Bank | Accession numbers |
| --- | --- | --- | --- | --- | --- |
| BH | BH | BH | Petroleum | MG-RAST | 4827355.3 |
| YPD | YPD | BH | Petroleum | MG-RAST | 4826970.3 |
| Control 1 | - | - | Photosynthetic | SRA | [SRX375283](https://www.ncbi.nlm.nih.gov/sra/SRX375283%5Baccn%5D) |
| Control 2 | - | Mineral medium | Sugarcane bagasse | SRA | [SRX474425](https://www.ncbi.nlm.nih.gov/sra/SRX474425%5Baccn%5D) |
| PDE | PDE | - | Petroleum | MG-RAST | 4643480.3 |
| LBL | LB | - | Petroleum | MG-RAST | 4643476.3 |
| BHLBL | LB | BH | Petroleum | MG-RAST | 4583773.3 |
| BHYPDL | YPD | BH | Petroleum | MG-RAST | 4583777.3 |

- Step not carried out to obtain the microbial consortium

| Table S2- General characteristics of metagenomes generated by shotgun genome sequencing | | | | | |
| --- | --- | --- | --- | --- | --- |
|  |  |  | Metagenome | | |
|  |  |  | PW | YPD | BH |
| Base pairs (bp) |  |  | 873,118,094 | 517,780,920 | 556,196,819 |
| Number of reads |  |  | 3,517,854 | 1,951,107 | 2,614,576 |
| Failed in quality control |  |  | 843,023 | 366,221 | 467,788 |
| Length average of reads (bp) |  |  | 248 | 265 | 213 |
| Ribosomal RNA genes |  |  | 13,028 | 12,569 | 22,773 |
| Annotated Proteins |  |  | 2,009,236 | 1,503,207 | 1,956,245 |
| Unknown Proteins |  |  | 446,751 | 69,110 | 159,197 |


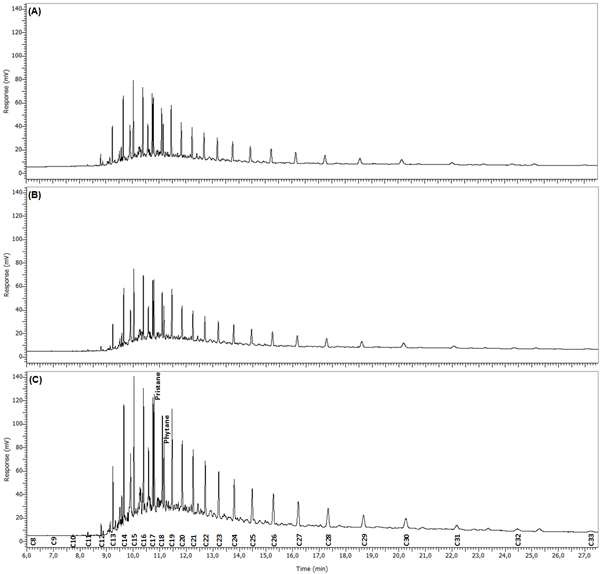


Figure S1- Chromatography of consortia. Chromatographic profile of biodegraded aliphatic hydrocarbons after the period of incubation by microbial consortia. (A) = consortium BH, (B) = consortium YPD and C = control.
